# Lack of Correlation between Accelerometers and Heart-Rate Monitorization during Exercise Session in Older Adults

**DOI:** 10.3390/ijerph17155518

**Published:** 2020-07-30

**Authors:** Laura Carbonell-Hernández, Diego Pastor, Alejandro Jiménez-Loaisa, Juan Arturo Ballester-Ferrer, Carlos Montero-Carretero, Eduardo Cervelló

**Affiliations:** Sport Research Center, Miguel Hernández University of Elche, 03202 Elche, Alicante, Spain; laura.carbonell@goumh.umh.es (L.C.-H.); alejandro.jimenezl@umh.es (A.J.-L.); juan.ballester05@goumh.umh.es (J.A.B.-F.); cmontero@umh.es (C.M.-C.); ecervello@umh.es (E.C.)

**Keywords:** heart rate, accelerometry, exercise session, intensity

## Abstract

Aging is increasing worldwide; hence, aging-related health is also more relevant. Well-programmed physical exercise is now an indispensable tool to achieve active aging and preserve older people’s health. Such “well-programmed” exercise requires efficient and useful tools to measure the activity. The objective of this study is to evaluate the effectiveness of accelerometers to estimate two different intensities of physical exercise in older people. Thirty-eight subjects (64.5 ± 5.3 years) were measured during two different sessions of physical exercise: one moderate in intensity, the other of low intensity. Heart rate and accelerometry were recorded and analyzed. The results showed that the two variables in the physical exercise sessions were not highly correlated, and that accelerometry did not seem useful to assess low-intensity sessions not based on walking.

## 1. Introduction

Evidence shows that the ratio of the aged population is growing worldwide due to declining birth rates and increased life expectancy [1]. Biologically, aging is an inevitable process that occurs throughout the life of species, with different evolution [2]. In humans, this process implies a progressive deterioration of physiological systems in the last years of life [3]. Aging is, therefore, a degenerative mechanism determined by genetic and environmental variables, in which lifestyle is strongly related to the development of the process [3]. Therefore, certain daily habits such as physical exercise, physical activity, smoking, or stress can regulate this process positively or negatively. 

As aging advances, the risks of fragility and disability increase [4]. Although aging cannot be stopped, its effects can be countered by certain behaviors or lifestyles. Physical exercise and physical activity are undoubtedly health- and disease-related variables [5]. Physical inactivity accentuates risk factors related to mortality and disease, such as cardiovascular disease, diabetes mellitus, or some cancers [5,6].

It is important to differentiate between physical activity and physical exercise, both of which are related to life-long health. On the one hand, physical activity refers to any movement produced by the muscle system that involves an increase in energy consumption; activities such as shopping, gardening, house cleaning, etc. increase people’s physical activity. On the other hand, physical exercise is a planned, structured, and repetitive physical activity aimed at improving one or more components of one’s physical condition [7]. Due to the risk of inactivity and the benefits of physical activity, the general physical activity recommendations of the American College of Sports Medicine (ACSM) are to increase physical activity through the habitual practice of well-scheduled physical exercise [7].

There are currently many physical exercise recommendations for the health of adults [8,9,10] and older people in particular [7]. Following the ACSM recommendations, older people should perform aerobic, strength, and flexibility exercise and, if required, balance/neuromotor exercise [7]. These different exercise modalities involve different activities during physical exercise sessions.

In sports, the importance of quantifying physical exercise is well known. The training load is measured to improve exercise prescription and to be more efficient in the training process, achieving better fitness goals [11]. In prescribed health-related physical exercise, individualized and tailored programs are currently being proposed to improve the impact of physical exercise on health [12]. This implies that physical exercise must be quantified by valid and reliable tools. Not only is accurate and objective information necessary, but such information must be accessible and, if possible, inexpensive to extend its use to the entire population. 

Questionnaires and diaries have commonly been used to measure physical activity or physical exercise, but they present certain problems of subjectivity and bias [11,13]. The Borg scale, used to measure the subjective perception of effort, is also commonly used to assess the intensity of exercise, but it does not always have a high relationship with the physiological changes produced during physical exercise [14]. Heart-rate (HR) monitoring is a useful tool for measuring the intensity of aerobic physical exercise [11], as HR and work intensity have a linear relationship when below the maximum oxygen consumption. 

Accelerometers have been used in recent years to measure people’s overall physical activity over long periods of time (days, weeks), showing their usefulness in adolescents [15], adults [16], and older people [17]. Accelerometry provides greater accuracy and precision than self-report measures or pedometers [18].

Accelerometers allow interpreting the time, intensity, and frequency of physical activity (e.g., sedentary, light, moderate, or vigorous activity) or its combined subcomponents (e.g., moderate to vigorous physical activity, usually expressed as MVPA) during specific periods of time. Accelerometers are easy to transport and use and can analyze a large amount of data [19].

Although accelerometry is considered a gold-standard measure to quantify people’s daily physical activity levels, this method has some limitations. This instrument cannot quantify the activity of upper body movements, aquatic activity or cycling, so certain activities are underestimated [20]. Moreover, accelerometers do not provide information about physical activity behavior [20]. Finally, the great variety of monitors, epoch lengths, and cut-off points selected are several factors that hinder comparisons between studies [21]. 

Accelerometers are useful to quantify overall physical activity. However, as general physical activity recommendations promote physical exercise to increase daily physical activity and health [7], it is necessary to ensure that the use of accelerometers will correctly quantify these scheduled and organized activities recommended for the general population.

The objective of this study is to evaluate the effectiveness of accelerometry to estimate two sessions of different physical exercise intensities in older people. For this purpose, heart-rate monitoring was used as a standard to correlate with accelerometer results. 

## 2. Materials and Methods

### 2.1. Sample

Thirty-eight subjects (13 males and 25 females; 64.5 ± 5.3 years old) participating in a physical exercise program twice a week were asked to take part in the experiment. Two sessions of this program, which were divided into two different intensities, were used for this purpose. The measurements were taken in the same place as their usual practice place, to maintain an ecological setting during measurement. The study was approved by the University ethical committee (2020.19.E.OIR; 2020.85.E.OIR) and was implemented following the Declaration of Helsinki. Written informed consent was obtained from the participants before any testing procedures.

### 2.2. Procedure

**Figure A1:**
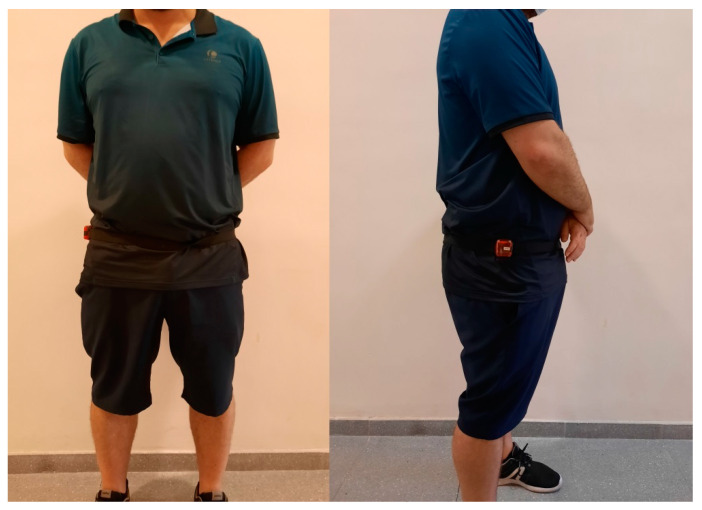
Actigraph gt3x position.

As participants had no previous experience in the use of these technologies (accelerometers and heart-rate monitors), they received a brief explanation of where to place the accelerometer and heart-rate monitor, how to adjust the elastic bands, and their use during physical activity practice. A monitor helped them place the devices. The accelerometers were placed on the right hip (Appendix A Figure A1), and the pulsometer monitor band was placed on the chest, three centimeters below the nipples, centered around the sternum. This was done just before the session began, and it took approximately 10 min each time to place all the devices and prepare them for measurement. 

Two different physical exercise sessions were performed with two objectives: a moderate-intensity aerobic session and a low-intensity session focused on balance/neuromotor exercises. The two sessions consisted of 10 min of warm-up, three blocks of 10 min of exercise, interspersing a minute of rest between blocks to hydrate, and ten minutes of cool-down at the end. The moderate-intensity session consisted of walking for 6 min, and then walking for 4 min while bouncing a basketball. The low-intensity session consisted of walking slowly on lines drawn on the ground, and performing balance and co-ordination games in place, along with some core exercises on the ground. The warm-up and cool-down were the same in both sessions. 

To ensure that the target intensity was maintained, the subjects’ maximum heart rate (HRmax) was estimated using Tanaka’s formula ((208−0.7) × Age) [22], and the subjects were monitored to maintain an HR between 65–75% of the HRmax during the moderate-intensity session and below 65% of the HRmax during the low-intensity session. The different HRs of the sessions were recorded and compared with a Student’s *t*-test. The moderate-intensity session presented a mean HR of M = 100.71 ± 9.71 beats per minute (bpm), and the low-intensity session presented a mean HR of 93.09 ± 10.72 bpm. The results showed a significant difference between the two sessions (*p* = 0.002). 

### 2.3. Instruments

#### 2.3.1. Heart-Rate Monitoring

**Figure A2:**
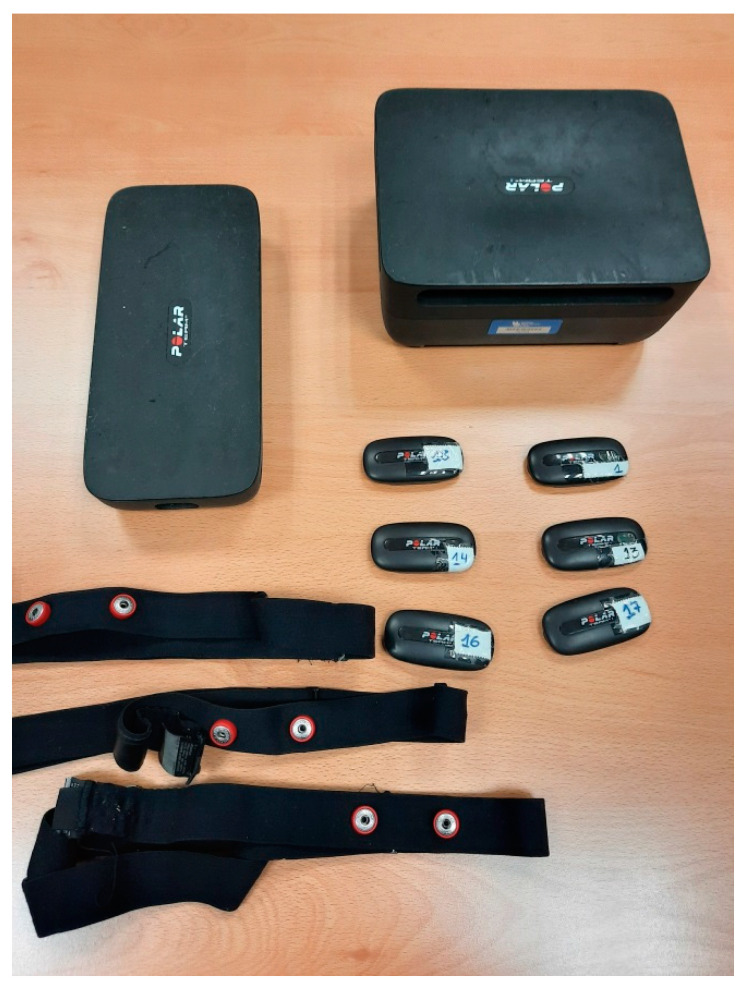
Polar Team 2 device.

HR was monitored with H10 pectoral bands using the Polar Team 2 instrument (Polar Team System, Polar Electro Oy, Kempele, Finland, Figure A2), which records the HR second-by-second directly from the chest. The second-by-second data were used to calculate the means of the minute-by-minute and the five-minute interval HR. 

#### 2.3.2. Accelerometers

ActiGraph GT3X monitor devices (ActiGraph, Pensacola, FL, USA) were used to obtain the accelerometry data. For the sake of precision and to obtain second-by-second information with the HR sensor, three-axis accelerometry (vector magnitude) was recorded, and a one-second epoch length was selected to record the data. Santos-Lozano et al.’s [23] cut-off points were selected to estimate energy expenditure, as these authors used the same device and the same population for these cut points. We recorded the second-by-second data for the analyses as well as the means of the minute-by-minute and 5-minute interval data. 

### 2.4. Statistical Analysis

Pearson correlations between variables were calculated. Statistical significance was placed at *p* = 0.05. Pearson *r*-values were classified following Mukaka’s criteria: values between 0.9 and 1.0 were considered very highly correlated, between 0.7 and 0.9 highly correlated, between 0.5 and 0.7 moderately correlated, and values between 0.3 and 0.5 were considered low correlations [24]. The minute-by-minute and five-minute interval accelerometry and heart-rate data were analyzed to calculate the linear regression between the two measurement tools. All statistical analyses were performed using the SigmaPlot 12.0 software (Systat Software Inc., San Jose, California, CA, USA). 

## 3. Results

### 3.1. Heart Rate and Accelerometry Correlations in Different Sessions

**Figure 1:**
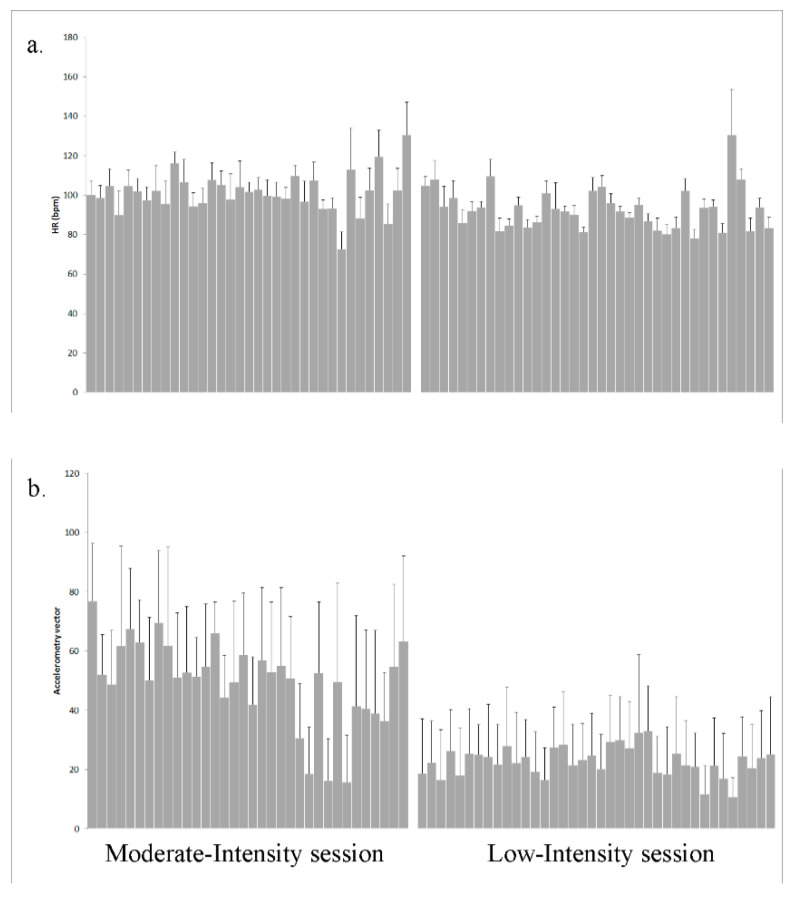
(**a**) Mean and standard deviation of the heart rate at each session for each of the subjects participating in the sessions. (**b**) Mean and standard deviation of the magnitude of the triaxial vector of the accelerometer of each session for each of the subjects participating in the sessions.

The average session results both of HR (beats per minute) and accelerometers (mean of the magnitude of the triaxial vector) of each of the subjects for the two sessions can be seen in Figure 1. Clear differences in accelerometry, as well as clear but lower differences in HR, can be observed.

Accelerometers show a subject’s activity second-by-second. Changes in cardiac response (HR) to physical activity are delayed compared to changes in intensity during the efforts. The 5-minute intervals, where this delay is assimilated in the interval, were therefore expected to show higher correlations with the accelerometry results. 

As can be seen in Table 1, the correlations were higher, and more significant, in the 5-minute intervals than in the minute-by-minute interval in the moderate-intensity session (Table 1). As expected, walking-based aerobic physical exercise showed significant correlations between HR and accelerometry, which were higher in the 5-minute interval (*r* = 0.519, *p* < 0.001) than in the minute-by-minute interval (*r* = 0.024, *p* = 0.297). Conversely, the low-intensity session, with slow movements and static exercises, did not correlate with any of the intervals. 

**Figure 2:**
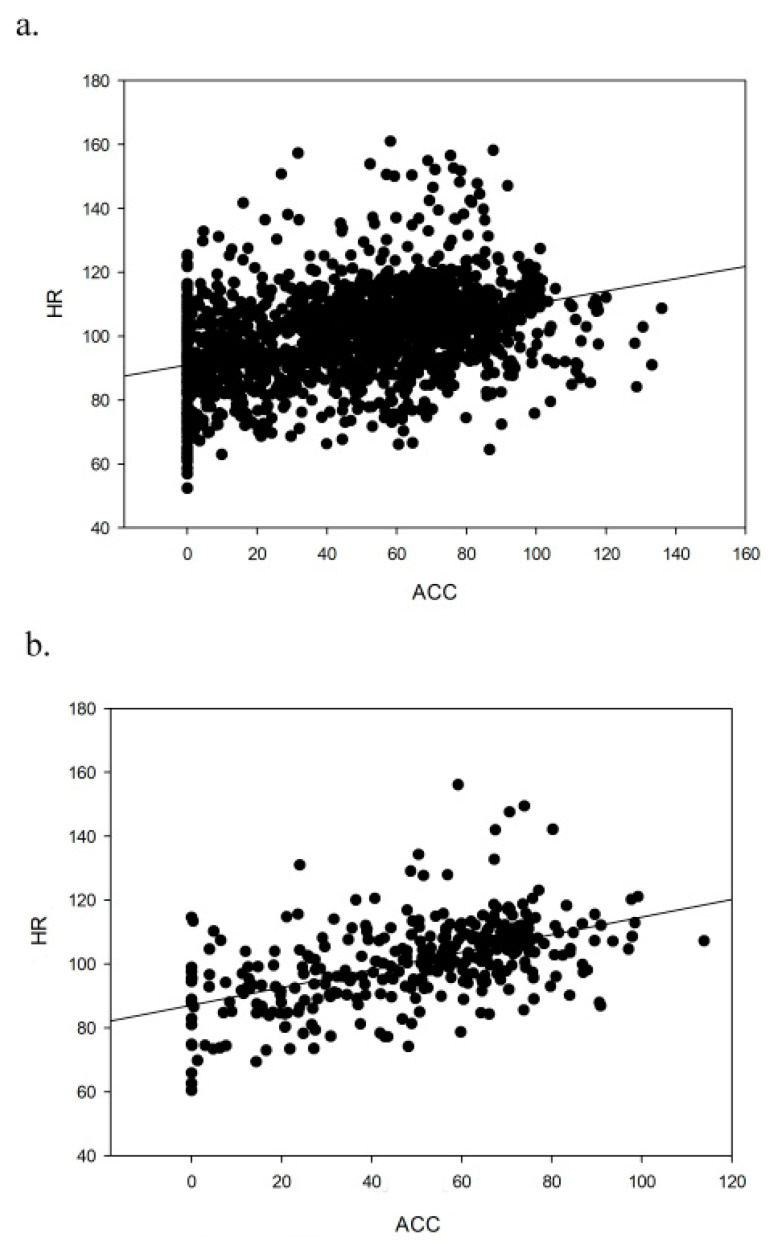
Linear regression of heart rate (beats per minute) and accelerometry (one-minute mean for the vector magnitude of the three axes) in the moderate-intensity aerobic session; (**a**) minute-by-minute, (**b**) 5-minute intervals.

Linear regression analysis showed a positive and significant relationship between accelerometry (ACC) and HR during the moderate-intensity aerobic session (Figure 2), both for the minute-by-minute analysis (ACC = −38.393 + (0.861 HR), Adjusted *R*^2^ (Adj Rsqr) = 0.165; Figure 2a) and for the five-minute intervals (ACC = −49.514 + (0.976 HR), Adj Rsqr = 0.267; Figure 2b).

**Figure 3:**
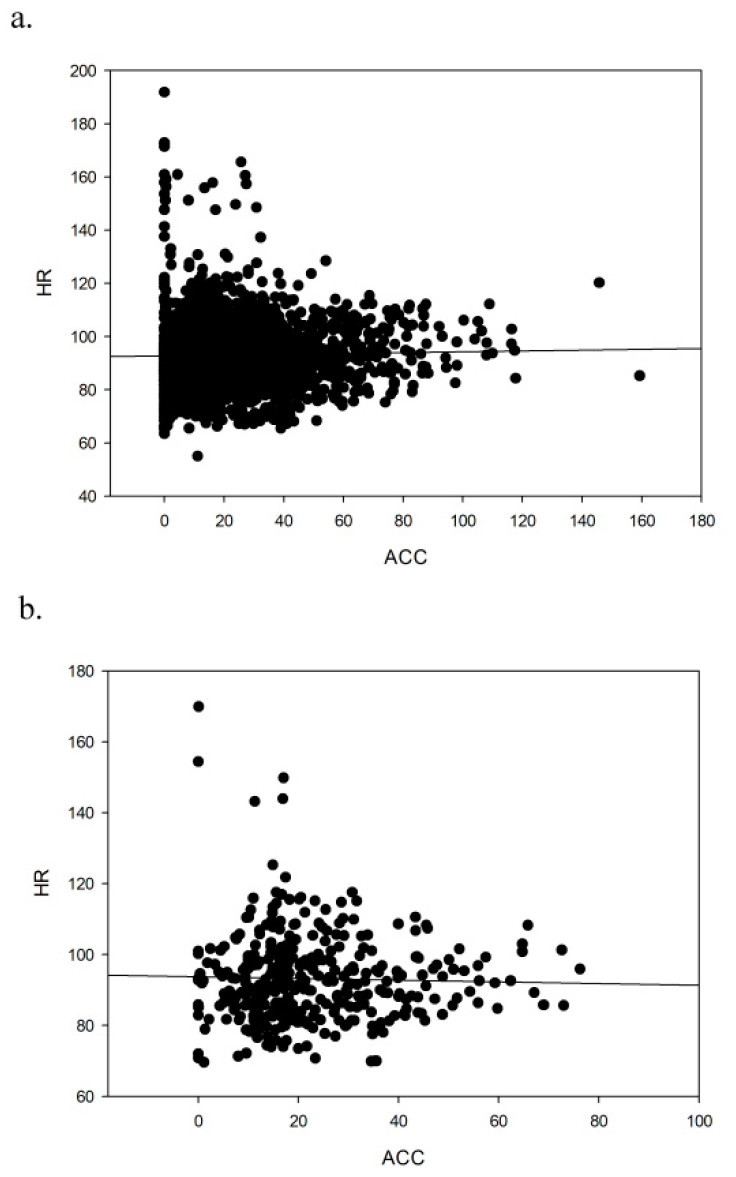
Linear regression of heart rate (beats per minute) and accelerometry (one-minute mean for the vector magnitude of the three axes) in the low-intensity session; (**a**) minute-by-minute, (**b**) 5-minute intervals.

In the low-intensity session with balance exercises, the linear regression showed no significant adjustments for any of the intervals (Figure 3) for either the minute-by-minute interval (ACC = 19.975 + (0.0376 HR), Adj Rsqr = 0.000047; Figure 3a) or the 5-minute interval (ACC = 26.110 − (0.0348 HR), Adj Rsqr = 0.000; Figure 3b).

### 3.2. Heart Rate and Energy Expenditure Estimation

Using Santos-Lozano’s cut-off points [23], the time spent in each session was calculated at different METs (Metabolic Equivalent Task defined as 3.5 mL O_2_ × kg^−1^ × min^−1^) of intensity: time under 3 METs, time between 3 and 6 METs, and time over 6 METs. 

When all the data obtained both from the moderate- and low-intensity sessions were conjointly analyzed, we observed a small negative correlation with the low-intensity modality (<3 METs) (Table 2). This is consistent because, the higher the subject’s HR, the less time they spend consuming low energy (<3 METs), as high HR requires high energy consumption. 

Interestingly, when analyzing the sessions separately, this correlation did not appear in the moderate-intensity session (Table 3), but it did emerge in the low-intensity session (Table 4). This is incongruous with the data shown in the linear regression, as the METs are calculated with the accelerometry data.

## 4. Discussion

The objective of this study was to evaluate the effectiveness of accelerometers in estimating physical activity during physical exercise sessions, as these sessions are generally recommended for increased activity and health [7]. This prescription of physical exercise includes sessions with different exercise modalities and different intensities [7], which should be as personalized as possible [12], making the quantification of such exercise an important parameter in the management of training programs. 

This study shows moderate correlations between HR and accelerometry in moderate-intensity aerobic sessions, but no correlation in low-intensity sessions based on balance. 

There is also an almost non-existent correlation between HR in a physical exercise session and the energy expenditure calculated with accelerometers during the same session. The moderate correlation between HR and accelerometry during moderate-intensity aerobic sessions may allow the use of accelerometers to monitor these activities, at least for the five-minute intervals. However, with these correlation values, it should be kept in mind that, when using the accelerometer, considerable information is lost about the physical activity carried out during this type of session. Low-intensity exercises based on balance are completely underestimated with the use of accelerometers. Other physical exercise modalities that are recommended, such as resistance training sessions, are probably also underestimated by this device. 

Accelerometers are often used to evaluate physical activity over extended periods of time, but not to quantify physical activity in particular physical exercise sessions [13]. However, to improve these levels of physical activity and health, it is recommended to perform physical exercise programs with different training-session modalities [7], and it should be taken into account that these activities will surely be underestimated when using the accelerometer to calculate individuals’ activity and energy expenditure. 

In this study, two main limitations were found to consider accelerometers as useful for controlling exercise programs in older people. First, accelerometers do not present a high correlation with moderate aerobic sessions. Second, the accelerometer is completely inadequate to assess the intensity of low-intensity sessions based on balance and neuromotor exercises. 

On the other hand, as Schrack et al. indicated in their study, we must take into account that activities such as walking are more intense for less functional subjects, [25] and this can hardly be taken into account by accelerometry. In fact, if less functionality involves more effort and more energy expenditure when walking or performing any other physical activity, we should have specific cut-off points for older people concerning their functionality, and not simply because of their age. 

Different cut-off points have been proposed to accurately measure the physical activity of older people. The cut-off points that Sanchez-Lozano [23] used in this article show a low deviation in low-intensity activities, such as those performed in this study, but higher deviations in other measures [26]. To our knowledge, there are no cut-off points for older people according to their functionality, and this would probably be necessary to gather more accurate information about physical activity programs. Likewise, it would be necessary to have different cut-off points for different types of physical exercise to more accurately estimate the physical activity carried out by older people when participating in organized physical exercise programs to improve their health. 

## 5. Conclusions

To conclude, we discourage the use of accelerometers to monitor physical exercise sessions in older people. Although its use seems appropriate to quantify daily physical [13] activity, we consider that it would be interesting for such quantifications to take into account that the physical activity performed in organized physical exercise programs is probably being underestimated by accelerometry. Nevertheless, more research is needed to investigate the role of accelerometers for these purposes.

## Figures and Tables

**Table 1:** Pearson correlations between HR and accelerometry data during different time intervals.

	Minute-by-Minute		5-Minute Intervals	
	MOD	LOW	MOD	LOW
*r*	0.407	0.024	0.519	0.106
*p*	<0.001	0.297	<0.001	0.583
*n*	1789	1891	343	357

MOD = Moderate-intensity aerobic session, LOW = Low-intensity balance session.

**Table 2:** Pearson correlations between HR and METs of both conditions.

HR	<3 METs	3–6 METs	6+ METs
*r*	−0.324	0.167	0.097
*p*	0.006	0.160	0.416
*n*	72	72	72

METs (Metabolic Equivalent Task defined as 3.5 mL O_2_ × kg^−1^ × min^−1^).

**Table 3:** Pearson correlations between HR and METs of moderate-intensity session.

HR	<3 METs	3–6 METs	6+ METs
*r*	0.057	−0.107	0.029
*p*	0.747	0.549	0.870
*n*	34	34	34

METs (Metabolic Equivalent Task defined as 3.5 mL O_2_ × kg^−1^ × min^−1^).

**Table 4:** Pearson correlations between HR and METs of low-intensity session.

HR	<3 METs	3–6 METs	6+ METs
*r*	−0.497	−0.089	0.018
*p*	0.002	0.592	0.911
*n*	38	38	38

METs (Metabolic Equivalent Task defined as 3.5 mL O_2_ × kg^−1^ × min^−1^).
